# *In Vivo* and *In Vitro* Analysis in Coronary Artery Disease Related to Type 2 Diabetes

**DOI:** 10.3389/fendo.2017.00209

**Published:** 2017-08-21

**Authors:** Teresa Infante, Ernesto Forte, Marco Aiello, Marco Salvatore, Carlo Cavaliere

**Affiliations:** ^1^IRCCS SDN, Naples, Italy

**Keywords:** type 2 diabetes, coronary artery disease, computed tomography coronary angiography, biomarkers, atherosclerosis

## Abstract

**Aim:**

The leading cause of morbidity and mortality in patients with type 2 diabetes mellitus (DM) is coronary artery disease (CAD), a condition often asymptomatic but severe in these patients. Although glucose metabolism impairment and oxidative stress are known actors in the endothelial dysfunction/remodeling that occurs in diabetic patients, the relationship between cardiovascular disorders and DM is not fully understood. We have performed both an *in vivo* imaging and *in vitro* molecular analysis to investigate diabetic-specific CAD alterations.

**Methods:**

Computed tomography coronary angiography (CTCA) was performed in a group of 20 diabetic patients with CAD (DM^+^CAD^+^), 20 non-diabetic with CAD (DM^−^CAD^+^), 10 diabetic non-CAD patients (DM^+^CAD^−^), and 20 non-diabetic healthy subjects (HS). Imaging quantitative parameters such as calcium score (Cascore), calcified plaque volume (CPV), non-calcified plaque volume (NCPV), total plaque volume (TPV), remodeling index (RI), and plaque burden were extracted for each CAD subject. Moreover, the expression levels of superoxide dismutase 2 (SOD2) and liver X receptor alpha (LXRα) genes were analyzed in the peripheral blood mononuclear cells, whereas hyaluronan (HA) concentrations were evaluated in the plasma of each subject.

**Results:**

Imaging parameters, such as Cascore, CPV, RI, and plaque burden, were significantly higher in DM^+^CAD^+^ group, compared to DM^−^CAD^+^ (*P* = 0.019; *P* = 0.014; *P* < 0.001, *P* < 0.001, respectively). SOD2 mRNA was downregulated, while LXRα gene expression was upregulated in DM^+^CAD^−^, DM^+^CAD^+^, and DM^−^CAD^+^ groups compared to HS (*P* = 0.001, *P* = 0.03, and *P* = 0.001 for SOD2 and *P* = 0.006, *P* = 0.008, and *P* < 0.001 for LXRα, respectively). Plasmatic levels of HA were higher in DM^−^CAD^+^, DM^+^CAD^−^, and DM^+^CAD^+^ groups, compared to HS (*P* = 0.001 for the three groups). When compared to DM^−^CAD^+^, HA concentration was higher in DM^+^CAD^−^ (*P* = 0.008) and DM^+^CAD^+^ (*P* < 0.001) with a significant difference between the two diabetic groups (*P* = 0.003). Moreover, HA showed a significant association with diabetes (*P* = 0.01) in the study population, and the correlation between HA levels and glycemia was statistically significant (ρ = 0.73, *P* < 0.001).

**Conclusion:**

In our population, imaging parameters highlight a greater severity of CAD in diabetic patients. Among molecular parameters, HA is modulated by diabetic CAD-related alterations while SOD2 and LXRα are found to be more associated with CAD but do not discriminate between diabetic and non-diabetic subgroups.

## Introduction

Type 2 diabetes mellitus (DM) is the most important risk factor for the onset of coronary artery disease (CAD), causing glucose metabolism impairment and endothelial dysfunction mediated by oxidative stress and inflammation (1). A complex network of signaling pathways is involved in these pathological processes leading to the development and progression of cardiac dysfunction. In response to myocardial damage, the heart undergoes a progressive anatomical and functional transformation known as “remodeling” (2).

Several imaging modalities have been used to detect CAD in diabetic patients including invasive coronary angiography, myocardial scintigraphy and dobutamine stress echocardiography (3). Even if invasive coronary angiography is the gold standard for identifying obstructive lesions, it only depicts the lumen of the vessel, greatly underestimating the burden of atherosclerosis (4). Myocardial scintigraphy and dobutamine stress echocardiography highlight perfusion defects (inducible ischemia and necrosis), but they lack a direct visualization of coronary arteries (5). Unlike these, computed tomography coronary angiography (CTCA) is a powerful diagnostic tool to rule out CAD thanks to its high negative predictive value (6). It allows quantification of atherosclerotic burden providing comprehensive information about the location, severity, and features of coronary atherosclerotic plaques and can be useful for risk stratification (7, 8).

Atherosclerosis is a multistage pathological condition involving an imbalanced lipid metabolism and immune response leading to a chronic inflammation of the arterial wall with the formation of the atherosclerotic plaque and consequent thickening of vessel wall and lumen stenosis (9, 10).

The first step of atherosclerosis is endothelial dysfunction; atherosclerotic lesions initiate in regions characterized by low shear stress resulting in an increase of adhesiveness of circulating monocytes to the vessel wall and subendothelial accumulation of low-density lipoprotein (LDL) (9). Common cardiovascular risk factors, such as smoking, diabetes, hypertension, and hypercholesterolemia, are causes of dysfunction endothelial (10). The LDL particles in the intima are susceptible to oxidation by reactive oxygen species or other enzymes released from inflammatory cells. Oxidized LDL triggers the expression of adhesion molecules and the secretion of chemokines by endothelial cells that drive the intimal infiltration by immune cells forming the so-called “fatty streaks” especially consisting of monocyte-derived macrophage-like foam cells. Subsequently, vascular smooth muscle cells migrate and proliferate into the site of lesion producing an excessive amount of connective tissue with the consequent formation of the fibroatheromatous plaque leading to thickening of vessel wall and stenosis of coronary lumen (9–11). One of the major issues in CAD diagnosis and management is that symptoms onset in the advanced state of disease. Indeed, most individuals show no manifestations for long time before the first onset of symptoms, often with a fatal event.

Oxidative stress is a key component in the development and progression of DM and its vascular complications such as CAD (12, 13). The onset and progression of CAD involves multiple cell types, and whole-blood gene expression profiling has the potential to provide information about dynamic changes in disease states and on underlying disease mechanisms (14).

Superoxide dismutase 2 (SOD2) is one of the major antioxidant defense systems against free radicals (15). Mutations or polymorphisms of SOD2 gene are associated with DM progression and complications, where the reduction of total antioxidant capacity and depletion of plasma antioxidants could be related to induced-oxidative stress damage (16–21).

Nuclear liver X receptors (LXR) comprise liver X receptor alpha (LXRα) and liver X receptor beta (LXRβ), which are key regulators of macrophage function, controlling transcriptional programs involved in lipid homeostasis and inflammation. The inducible LXRα is highly expressed in macrophages, liver, adrenal gland, intestine, adipose tissue, lung, and kidney, whereas LXRβ is ubiquitously expressed (22). LXRs are involved in the regulation of cholesterol metabolism fundamental for the pathogenesis of CAD and inhibit atherogenesis, inflammation and autoimmune reactions (22). Furthermore, an additional role of LXRs is to contribute to glucose homeostasis, demonstrating potent glucose-lowering and insulin-sensitizing effects (23, 24). Despite extensive research in the field of LXR biology, however, very little is known about the regulation of expression and activity of these receptors.

Hyaluronan (HA) is present in low amount in normal blood vessels but increases in vascular diseases as well as in DM (25). It seems to have an important role in diabetic angiopathy (26–28) and is associated with an increased risk for developing CAD also in non-diabetic patients (29). HA is increased in vascular plaques, and its high metabolism causes their destabilization (30). Furthermore, the fragmentation of HA triggers inflammatory processes and activates leukocytes to produce superoxide radical causing oxidative stress (31).

To date, studies integrating parameters calculated by CTCA and biological markers in DM patients have been focused on the association between CTCA findings (mostly coronary artery calcium) and biological markers of inflammation (IL-6, IL-1β, TNF-α, hs-CRP, and YKL-40) and endothelial dysfunction (sVCAM-1, sICAM-1, and sICAM-3) (32–36). There are no data about the association between imaging parameters and gene expression profiling in DM.

In this study, we have analyzed the three above mentioned molecular markers that underlie important steps of the atherosclerotic process: endothelial dysfunction, oxidative stress, lipid homeostasis, and inflammation. In this regard, we have analyzed SOD2 and LXRα gene expression and HA plasmatic concentrations in a group of 20 diabetic patients with known CAD (DM^+^CAD^+^), 20 non-diabetic patients with CAD (DM^−^CAD^+^), 10 diabetic non-CAD patients (DM^+^CAD^−^), and 20 non-diabetic healthy subjects (HS). Furthermore, the purpose of our study was to investigate diabetic-specific CAD alterations using both quantitative imaging parameters derived from CTCA and molecular biomarkers.

## Materials and Methods

### Patient Recruitment

Computed tomography coronary angiography was performed in 20 DM^+^CAD^+^ patients, 20 DM^−^CAD^+^ patients, 10 DM^+^CAD^−^ patients, and 20 HS referred to our institution for suspected CAD. All clinical characteristics such as laboratory parameters, presence of cardiovascular risk factors, and medical history were accurately recorded.

Diabetes was defined as treatment with drugs or fasting blood glucose ≥126 mg/dL. Dyslipidemia was defined as treatment with drugs or fasting serum total cholesterol ≥240 mg/dL, or LDL cholesterol ≥140 mg/dL, or high-density lipoprotein cholesterol <40 mg/dL, or triglyceride ≥150 mg/dL. Hypertension was defined as treatment with drugs or systolic blood pressure (SBP) ≥140 mmHg or diastolic blood pressure (DBP)≥ 90 mmHg. Anthropometrical measurements including body weight and height were recorded and body mass index (BMI) was calculated. Blood pressure and resting heart rate were measured after ≥5 min rest with a sphygmomanometer. Physical activity in HS and patients was evaluated according to the WHO guidelines for adults 18–65 aged; specifically the performance of at least 150 min of moderate-intensity aerobical physical activity per week [50–70% of maximum heart rate (MHR)] or at least 75 min of vigorous-intensity aerobic physical activity throughout the week (70–80% MHR) (37). None of the recruited subjects had physical disabilities.

Patients with known history of cancer, cardiomyopathy, active infections, chronic or immune-mediated diseases, renal failure, hepatic failure, and not suitable for cardiac imaging (atrial fibrillation, arrhythmia, or pre-scan heart rate greater than 65 bpm) were excluded from the study to avoid confounding effects due to other variables.

### Sample Collection

Peripheral venous blood samples were collected after a 12 h overnight fasting immediately before i.v. cannulation for CTCA examination. All tubes were centrifuged within 30 min of collection at 1,900 *g* for 10 min at 4°C to separate plasma and cellular components. Aliquots of plasma were transferred into cryostat tubes and stored at −80°C until analysis. PBMNCs were isolated by Ficoll gradient using HISTOPAQUE-1077 (Sigma Diagnostics, MO, USA) and frozen at −80°C until total RNA extraction. All biological samples were stored at the IRCCS SDN Biobank (38). The study and the protocol were approved and reviewed by the institutional ethics committee (IRCCS Fondazione SDN, protocol no. 7-13). The study was performed in accordance with the ethical standards of the institutional ethics committee and with the Helsinki Declaration. A written informed consent was obtained from all subjects enrolled.

### CT Angiography Protocol and Image Analysis

Computed tomography coronary angiographies were performed on a CT scanner (Discovery CT750 HD, GE Healthcare), with a 64 mm × 0.625 mm collimation, 350 ms rotation time, and 228 ms temporal resolution. A prospective ECG-triggered scan without contrast medium was used for calcium score (Cascore) evaluation followed by a retrospective scan with ECG tube current modulation. Contrast enhancement was obtained by a bolus tracking technique with scan starting when a region of interest placed in the ascending aorta at the pulmonary bifurcation reached a threshold of 150 Hounsfield unit (HU). Contrast material (iomeprol 400 mg I/mL, Iomeron 400, Bracco, Milan, Italy) was injected at 5–6 mL/s through an 18-gauge intravenous antecubital catheter and was followed by saline solution at the same flow. Tube voltage and contrast agent volume were adapted to patient anatomical features such as BMI, calcifications, or stents. Images were reconstructed with a section thickness of 0.625 mm and an increment of 0.4 mm; standard and sharp reconstruction filter kernels were used; an additional sharper convolution kernel was used in patients with stents or calcification. The best data set was chosen according to the phase of the cardiac cycle with lower artifacts and coronary motions. Images were sent to a dedicated offline workstation (GE Advantage workstation 4.6, GE Healthcare) where MIP, cMPR, and 3D volume rendering were generated. Cascore was calculated by the SmartScore tool to obtain the Agatston score. Total plaque volume (TPV), non-calcified plaque volume (NCPV), calcified plaque volume (CPV), and total lumen volume were measured for the major coronaries using the HU cutoff values reported in Ref. (39). The resulting values were summed to determine a per-patient plaque volume. Total vessel volume was determined summing TPV and total lumen volume. Plaque burden was obtained dividing TPV by total vessel volume (40). The remodeling index (RI) was calculated by dividing the cross-sectional vessel area at the site of maximum luminal narrowing including plaque by the cross-sectional vessel area in the most proximal atherosclerotic free segment chosen as reference (41). The total number of coronary artery segments exhibiting plaque (NSP) was determined according to the modified American Heart Association 16-segment classification (42) for each patient (less or more than 8 segments affected). Significant coronary stenosis was defined as a decrease in the luminal diameter of >50% in one or more of the major coronary arteries; the total number of coronaries (NCS) with significant stenosis was calculated for each patient (less or more than one stenotic vessel). All scans were analyzed by two experienced, independent radiologists; therefore, a consensus interpretation was arrived to obtain a final coronary CT diagnosis according to the international SCCT guidelines (43).

### RNA Extraction and Reverse Transcription

Total RNA was isolated from PBMCs using TRIzol Reagent (Thermo Fischer Scientific, USA) as previously described (44). The quantity and quality of RNA were measured using the NanoDrop 1000 (Thermo Fischer Scientific, USA). Total RNA (0.5 µg) was reversed transcribed (RT) using the SuperScript^®^ III First-Strand Synthesis SuperMix for qRT-PCR (Thermo Fischer Scientific, USA) according to the protocol of the manufacturer. The RT was performed using the Bio-Rad iCycler Thermal Cycler with the following protocol: incubation at 25°C for 10 min (primer annealing), 42°C for 30 min (cDNA synthesis), and 85°C for 5 min (termination of cDNA synthesis). Immediately after, the samples were cooled down and stored at −20°C.

### Quantitative Real-time PCR

The optimal reference genes for the study were selected as previously reported (45). Gene expression was quantified on the MyiQ™ Single-Color Real-Time PCR Detection System (Bio-Rad Laboratories, USA). Primers pairs were designed through OLIGO 6.7 program, and their specificity was verified with the BLAST program for test of sequence homology, a test for secondary structures and optimization of multiplex setup. All primers were purchased from Life Technologies. All samples were run in triplicate for genes of interest and reference genes using 1 µL of cDNA and iQ™ SYBR^®^ Green Supermix (Bio-Rad Laboratories, USA) in a 25 µL final volume reaction. The thermal profile employed was 3 min of initial step of denaturation at 95°C followed with denaturation for 15 s at 95°C, annealing at 60°C for 30 s, and elongation at 72°C for 30 s for 40 cycles. Melt curve analysis was performed to verify a single product species. Relative expression (fold change) was calculated by the 2^−ΔΔCT^ method (46). Mean and SE were determined by averaging relative expression levels across three independent experiments, each determined in triplicate.

### HA Measurement

Plasmatic levels of HA were determined by enzyme-linked immunosorbent assay (ELISA) using Quantikine Hyaluronan Immunoassay kit (DHYAL0) (R&D Systems, Abingdon, UK), in accordance with the protocol supplied by the manufacturer. Briefly, samples were incubated with HA binding protein coated on microplates for 2 h at room temperature. After incubation, the microplates were washed five times with wash buffer, and further incubated with 100 µL of peroxidase labeled HA binding protein for 2 h at room temperature. After incubation, the microplates were again washed five times, and further incubated with 100 µL of peroxidase substrate for 30 min at room temperature in a dark room. The reaction was stopped by the addition of 100 µL of stop solution. The optical density of each well was determined using a microplate reader set to 450 nm within 30 min. HA concentration in each sample was calculated using the standard curve obtained with the purified HA solutions, included in the kit as references.

### Statistical Analysis

Statistical analysis was performed using R Core Team (version 3.03 Austria, Vienna). Continuous variables were expressed as mean ± SD or as median (1 quartile and 3 quartile). Data were tested for normality through the Shapiro–Wilk test and for homoscedasticity through the Levene test. For comparison between two groups, *t*-test was used if gaussianity was met; otherwise the Mann–Whitney *U* test was chosen. For comparison among four groups, the one-way analysis of variance was used if both gaussianity and homoscedasticity were met; otherwise the Kruskal–Wallis test was chosen. In case of statistical significance, the Tukey–Kramer test and the Conover test were used for multiple comparisons as parametric and non-parametric test, respectively. Categorical variables were expressed as percentage and were compared using the Fisher’s exact test. The Spearman correlation test was performed to assess linear relationship between variables; in case of binary variables, the association was tested by the Wilcoxon rank sum test. A *P* < 0.05 was considered for statistical significance (rounded to the third decimal place).

## Results

### Clinical Characteristics of Study Groups

The baseline demographic and clinical characteristics of the study population are summarized in Table 1. Heart rate was significantly different between HS and DM^+^CAD^+^ (*P* < 0.01) and HS and DM^−^CAD^+^ (*P* < 0.01) since only 10% of HS was in treatment with beta blocker agents, while no statistical significance was found between both CAD groups and DM^+^CAD^−^. Considering the metabolic markers, glycemia was significantly higher in DM^+^CAD^+^ and DM^+^CAD^−^ patients compared to HS (*P* < 0.01 and *P* < 0.001, respectively) and DM^−^CAD^+^ subjects (*P* < 0.01 and *P* < 0.001, respectively). Of diabetic patients, in DM^+^CAD^+^ group, 16% were insulin users, 64% were in treatment with antihyperglycemic agents, and 20% were not in treatment; in DM^+^CAD^−^ group, the percentage of treatments were, respectively, 10% for insulin, 80% for antihyperglycemic drugs, and 10% were not treated. Total cholesterol, LDL- and HDL-cholesterol plasmatic concentrations did not significantly differ among the four groups, reflecting the effects of statin therapy to which 73.68% of DM^+^CAD^+^, 50% of DM^−^CAD^+^, 40% of DM^+^CAD^−^, and 5% of HS were subjected. Furthermore, SBP and DBP were not statistical different among the groups (*P* = 0.50 and *P* = 0.52, respectively). In this regard, hypertensive patients were in treatment with beta blocker agents (*P* = 0.008), calcium channel blockers (*P* = 0.68), and ACE inhibitors (*P* = 0.06).

**Table 1:** Clinical parameters of patients and healthy subjects.

Clinical parameters	HS	DM^+^CAD^−^	DM^+^CAD^+^	DM^−^CAD^+^	*P* value^a^
Age	58 ± 8.37	60.8 ± 13.5	61.24 ± 10.47	64.4 ± 9.33	0.07
BMI	26.9 ± 3.62	28.94 ± 4.2	29.36 ± 5.43	27.73 ± 3.14	0.07
SBP (mmHg)	119.38 ± 11.16	122 ± 14.7	126.25 ± 19.20	118.75 ± 7.91	0.50
DBP (mmHg)	75.63 ± 6.23	77 ± 5.81	74.67 ± 10.36	78.75 ± 3.54	0.52
Heart rate (bpm)	67.86 ± 11.09	60.20 ± 6.15	56.00 ± 6.50	54.68 ± 6.49	0.005
Ejection fraction (%)	57.50 ± 3.54	55.78 ± 6.07	51.46 ± 9.90	56.00 ± 5.00	0.41
Glycemia (mg/dL)	93.71 ± 10.26	126.90 ± 14.43	132.43 ± 26.15	97.90 ± 12.17	0.006
Azotemia (mg/dL)	36.91 ± 7.44	37.8 ± 9.15	39.84 ± 16.04	38.48 ± 11.84	0.71
Creatinine (mg/dL)	0.88 ± 0.17	1 ± 0.19	1.03 ± 0.19	1.04 ± 0.17	0.05
Sex (M)	60%	60%	70%	75%	0.50
CAD familiarity	45%	40%	60%	60%	0.51
Smoke	25%	30%	15%	35%	0.75
Hypertension	45%	70%	75%	55%	0.04
Dyslipidemia	35%	70%	75%	45%	0.05
Physical activity	25%	20%	200%	25%	0.80
Total cholesterol (mg/dL)	187.35 ± 31.55	160.7 ± 72.45	160.75 ± 65.95	172.80 ± 47.31	0.51
LDL-c (mg/dL)	133.86 ± 26.47	87.24 ± 38.45	83.50 ± 68.59	107.60 ± 43.71	0.31
HDL-c (mg/dL)	55.25 ± 19.55	48.27 ± 15.50	48.50 ± 12.40	41.40 ± 8.02	0.45
Tryglycerides (mg/dL)	123.57 ± 52.99	135.28 ± 55	150.40 ± 66.54	119.25 ± 53.21	0.70
**Medical treatments**					
Beta blocker agents (%)	10%	40%	52.63%	52.63%	0.008
Calcium channel blockers (%)	20%	10%	10.53%	21.05%	0.68
ACE inhibitors (%)	15%	30%	42.11%	21.05%	0.06
Statins (%)	5%	40%	73.68%	50%	0.008
Antiplatelets agents (%)	5%	10%	78.95%	52.63%	<0.001
**Diabetic medications**					
Oral hypoglicemic (%)		80%	64%		0.04
Insulin (%)		10%	16%		0.23
No treatment (%)		10%	20%		0.05

*^a^Comparison among HS, DM^+^CAD^−^, DM^+^CAD^+^, and DM^−^CAD^+^*.

*SBP, systolic blood pressure; DBP, diastolic blood pressure; CAD, coronary artery disease; LDL, low-density lipoprotein; BMI, body mass index*.

### Imaging Parameters

**Figure 1:**
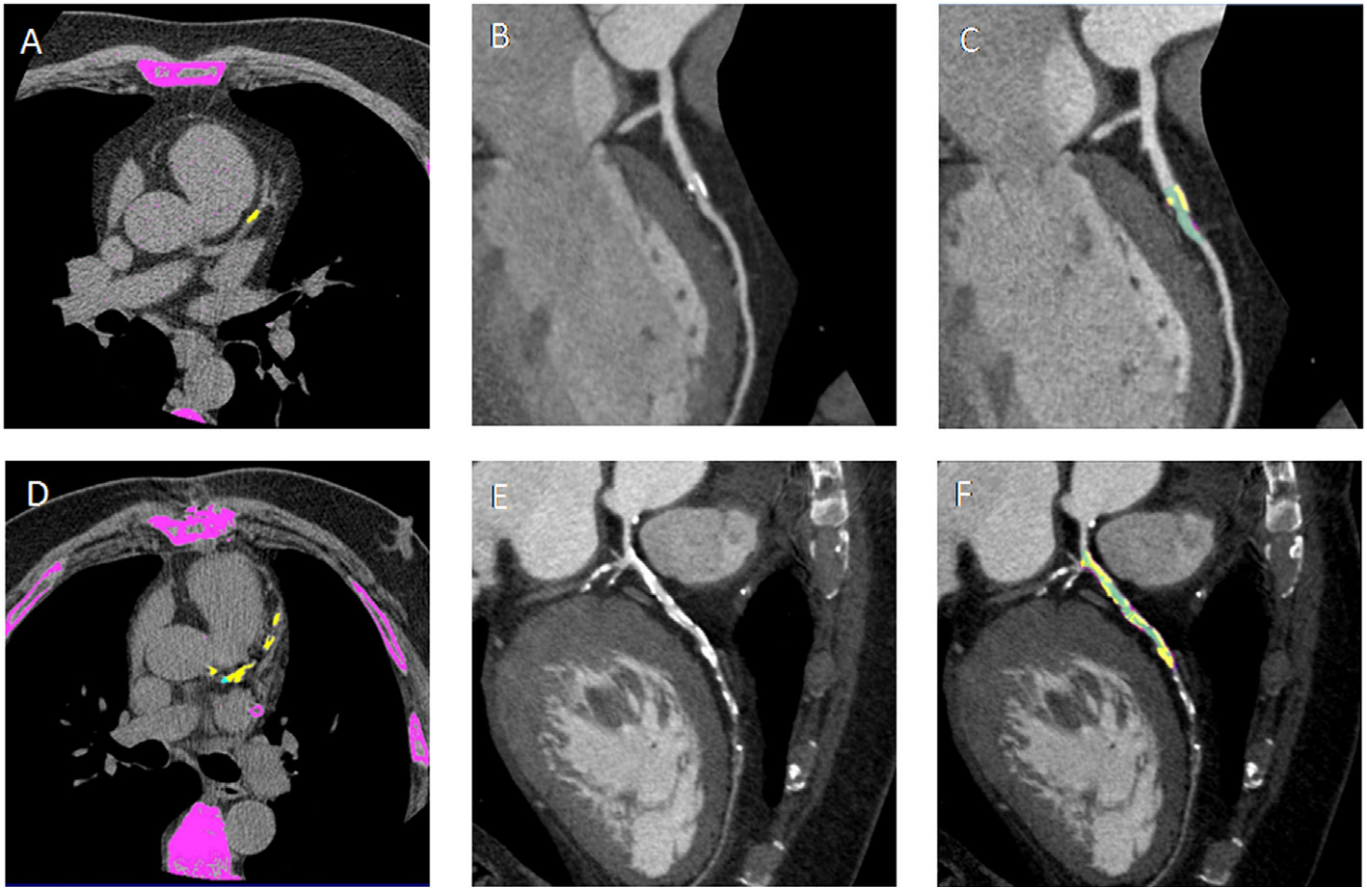
**(A,D)** Non-contrast enhanced images showing calcium deposits (yellow) on the left descending coronary artery (LAD) in a non-diabetic CAD patient (DM^−^CAD^+^) and in a diabetic CAD patient (DM^+^CAD^+^), respectively. **(B,E)** cMPR of LAD is provided for DM^−^CAD^+^ and DM^+^CAD^+^. **(C,F)** Plaque characterization: the calcific (yellow) and non-calcific (pink) components of the plaque are highlighted; the vessel lumen is represented in green. DM^+^CAD^+^ displayed significantly higher coronary calcium values compared to DM^−^CAD^+^.

**Figure 2:**
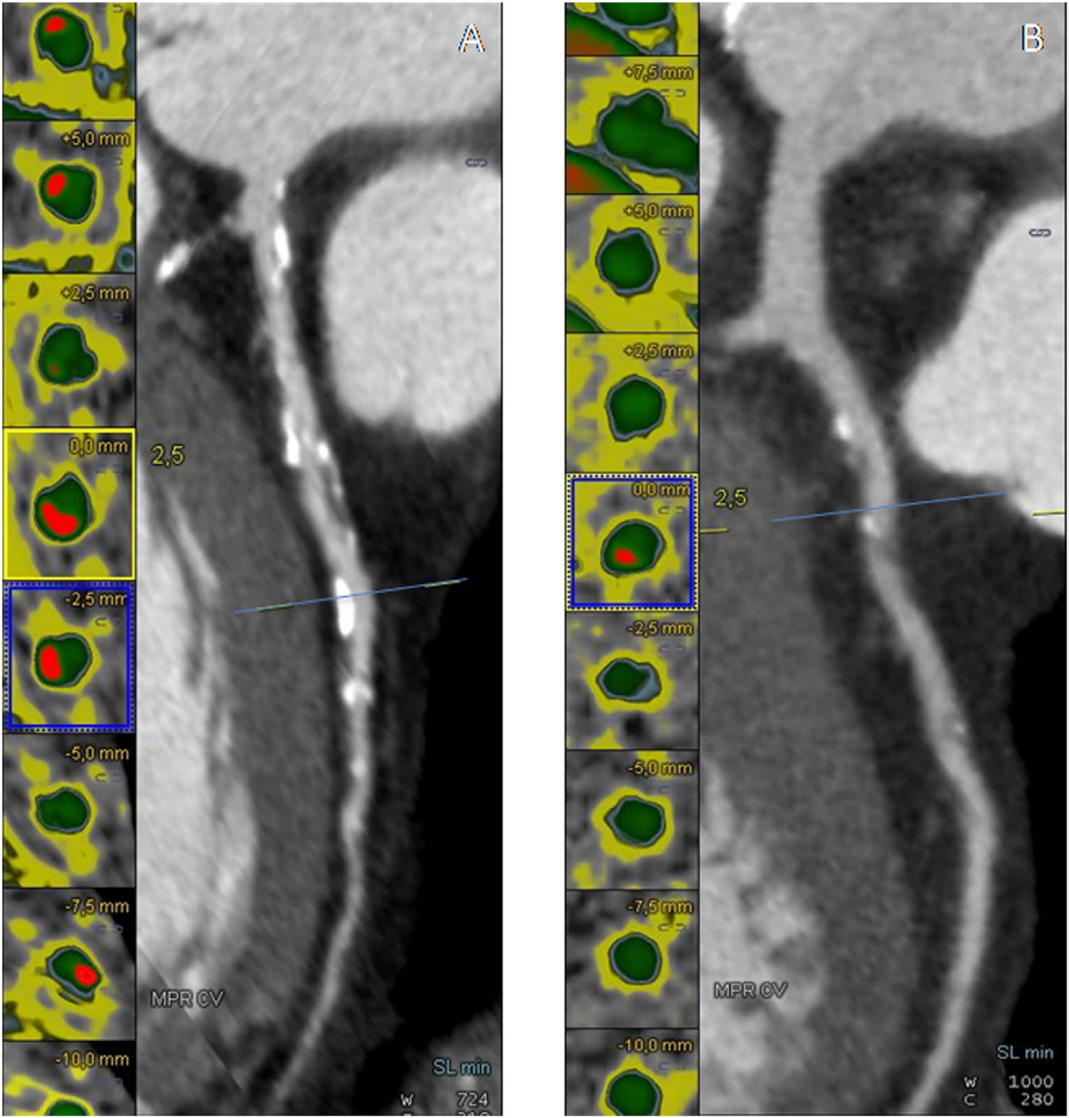
**(A,B)** Cross-sectional view and cMPR of the left descending coronary artery (LAD) in a non-diabetic CAD patient (DM^−^CAD^+^) and in a diabetic CAD patient (DM^+^CAD^+^). In cross-sectional images, the vessel lumen is represented in green whereas the calcific component of the plaque is red.

There was significant difference between DM^−^CAD^+^ and DM^+^CAD^+^ according to NCS and NSP (*P* = 0.026, *P* = 0.04, respectively). Cascore was significantly higher in DM^+^CAD^+^ compared to DM^−^CAD^+^ (Figures 1 and 2): 1,068.7 (517.2–2,086.85) vs 214.05 (72.98–970.15) *P* = 0.019. As regards plaque characterization, CPV was significantly higher in DM^+^CAD^+^ [105.85 (51.2–341.73) mm^3^] compared to DM^−^CAD^+^ [42 (7.2–105.9) mm^3^] *P* = 0.014, but there was no significant difference according to NCPV and TPV: 519.85 (411.93–1,064.85) mm^3^ for DM^+^CAD^+^ and 421.85 (240.10–689.58) mm^3^ for DM^−^CAD^+^ *P* = 0.37 and 688.95 (470.05–1,436) mm^3^ for DM^+^CAD^+^ vs 454.45 (257.78–820.83) mm^3^ for DM^−^CAD^+^ *P* = 0.16, respectively. RI was 1.40 ± 0.24 for DM^+^CAD^+^ and 1 ± 0.19 for DM^−^CAD^+^ *P* < 0.001, and plaque burden was 0.45 ± 0.14 for DM^+^CAD^+^ and 0.27 ± 0.15 for DM^−^CAD^+^ *P* < 0.001. Results are summarized in Table 2.

**Table 2:** Imaging parameters.

Imaging parameters	DM^+^CAD^+^	DM^−^CAD^+^	*P* value^a^
Number of coronaries with stenosis[Table-fn tfn3]	44.4%^c^	10%^c^	0.026
NSP^b^	78%^d^	45%^d^	0.04
Calcium score^b^	1,068.7 (517.2–2,086.85)	214.05 (72.98–970.15)	0.019
Calcified plaque volume (mm^3^)^b^	105.85 (51.2–341.73)	42 (7.2–105.9)	0.014
Non-calcified plaque volume (mm^3^)^b^	519.85 (411.93–1,064.85)	421.85 (240.10–689.58)	0.37
Total plaque volume (mm^3^)^b^	688.95 (470.05–1,436)	454.45 (257.78–820.83)	0.16
Remodeling index	1.40 ± 0.24	1 ± 0.19	<0.001
Plaque burden	0.45 ± 0.14	0.27 ± 0.15	<0.001

*^a^Comparison among DM^+^CAD^+^ and DM^−^CAD^+^*.

*^b^Data are expressed as median (1 quartile–3 quartile)*.

*^c^Patients with >1 coronary stenotic vessels*.

*^d^Patients with >8 coronary segments exhibiting plaque*.

In our population, RI highly correlated with plaque burden (ρ = 0.65, *P* < 0.001). Cascore showed a positive correlation with NCPV (ρ = 0.83, *P* < 0.001), CPV (ρ = 0.96, *P* < 0.001), TPV (ρ = 0.88, *P* < 0.001), and plaque burden (ρ = 0.60, *P* < 0.001). Moreover, a significant correlation was found between plaque burden and NCPV (ρ = 0.57, *P* < 0.001), CPV (ρ = 0.64, *P* < 0.001), and TPV (ρ = 0.60, *P* = 0.001).

### Gene Expression Profiling

**Figure 3:**
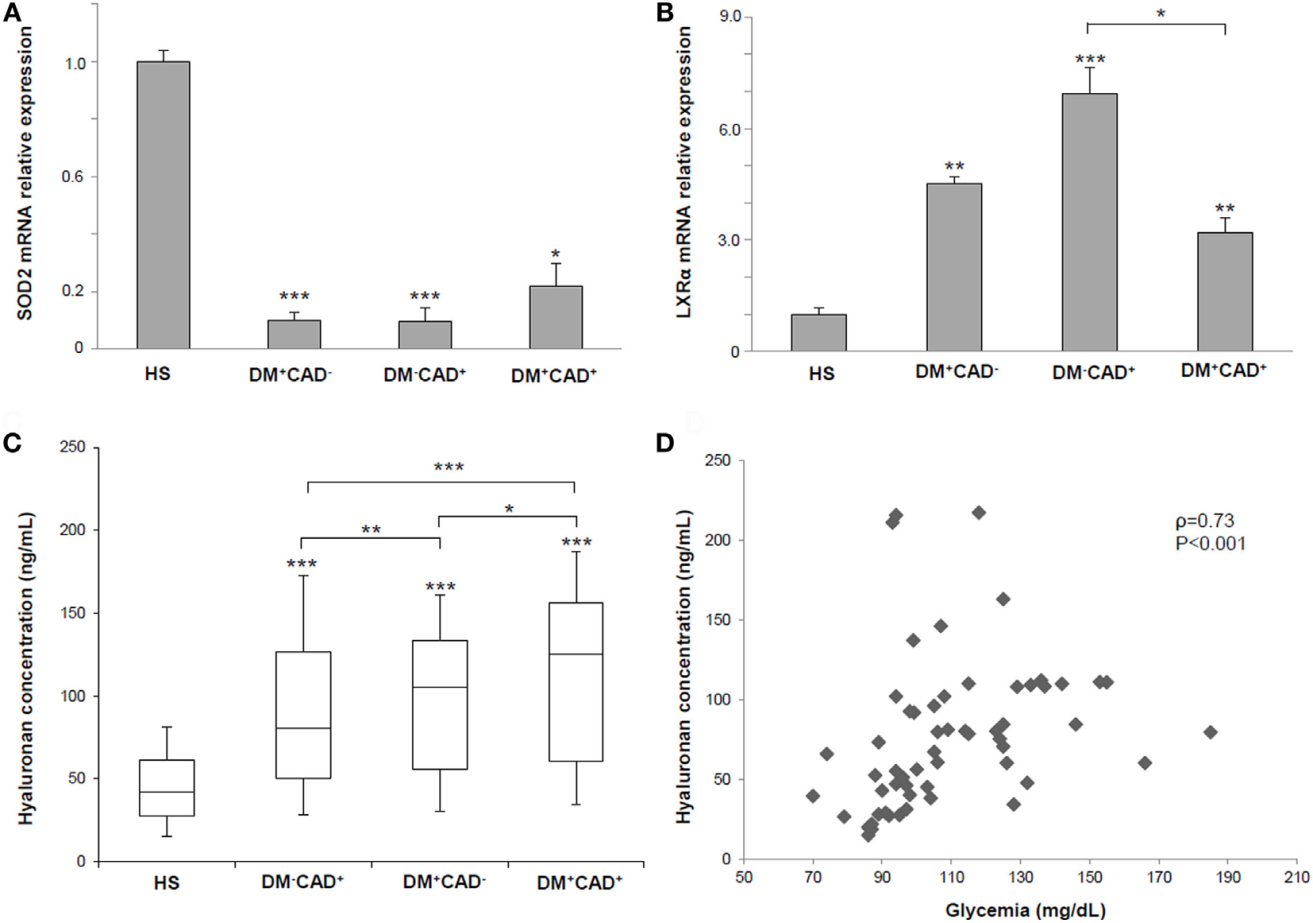
**(A)** Superoxide dismutase 2 (SOD2) mRNA relative expression in PBMNCs of healthy subjects (HS), diabetic non-CAD patients (DM^+^CAD^−^), non-diabetic CAD patients (DM^−^CAD^+^), and diabetic CAD patients (DM^+^CAD^+^). **(B)** Liver X receptor alpha (LXRα) gene expression in PBMNCs of HS, DM^+^CAD^−^, DM^−^CAD^+^, and DM^+^CAD^+^. **(C)** Median plasma hyaluronan (HA) concentrations (ng/mL) in HS, DM^+^CAD^−^, DM^−^CAD^+^, and DM^+^CAD^+^ (*P < 0.05; **P < 0.01; ***P < 0.001). When not specified, significance is referred to the comparison vs HS. **(D)** Correlation between HA levels and glycemia in HS, DM^+^CAD^−^, DM^−^CAD^+^, and DM^+^CAD^+^ patients (ρ = 0.73, *P* < 0.001).

We evaluated, by quantitative real-time PCR, SOD2 and LXRα gene expression in PBMCs from our population (Table 3). For both genes, ΔCT was computed and compared between the four groups. Molecular analysis showed that SOD2 mRNA was downregulated in DM^+^CAD^−^ (ΔCT = 5.70 ± 3.28; fold change = 0.10 ± 0.03; *P* = 0.001), DM^+^CAD^+^ (ΔCT = 4.57 ± 3.56; fold change = 0.22 ± 0.08; *P* = 0.03), and DM^−^CAD^+^ (ΔCT = 5.75 ± 3.04; fold change = 0.10 ± 0.05; *P* = 0.001) compared to HS (ΔCT = 2.36 ± 2.61), with no statistically significant difference between the two CAD groups (with and without DM) (Figure 3A). LXRα gene expression was significantly upregulated in DM^+^CAD^−^ (ΔCT = 2.77 ± 1.36; fold change = 4.51 ± 0.20 *P* = 0.006), DM^+^CAD^+^ (ΔCT = 3.27 ± 1.79; fold change = 3.19 ± 0.42; *P* = 0.008), and DM^−^CAD^+^ (ΔCT = 2.15 ± 1.16; fold change = 6.93 ± 0.70; *P* < 0.001) compared to the HS (ΔCT = 4.94 ± 2.14), with a significant difference between the two CAD groups (*P* = 0.03) (Figure 3B). No statistically significant correlation was found between SOD2 and Cascore (ρ = −0.04, *P* = 0.81), NCPV (ρ = −0.03, *P* = 0.85), and TPV (ρ = −0.02, *P* = 0.89) as well as between LXRα and Cascore (ρ = 0.13, *P* = 0.44), NCPV (ρ = 0.10, *P* = 0.55), and TPV (ρ = 0.10, *P* = 0.57).

**Table 3:** Molecular parameters.

Molecular parameters	HS	DM^+^CAD^−^	DM^+^CAD^+^	DM^−^CAD^+^	*P* value^a^
Superoxide dismutase 2	2.36 ± 2.61	5.70 ± 3.28	4.57 ± 3.56	5.75 ± 3.04	0.009
Liver X receptor alpha	4.94 ± 2.14	2.77 ± 1.36	3.27 ± 1.79	2.15 ± 1.16	0.005
Hyaluronan	46.90 ± 23.79	105.56 ± 18.13	120.74 ± 21.17	90.05 ± 35.11	<0.001

*^a^Comparison among HS, DM^+^CAD^−^, DM^+^CAD^+^, and DM^−^CAD^+^*.

### Comparison of HA Levels

In the HS group, mean concentration of plasma HA was 46.90 ± 23.79 ng/mL. Compared with HS, HA concentrations were higher in DM^−^CAD^+^ (90.05 ± 35.11 ng/mL; *P* = 0.001), DM^+^CAD^−^ (105.56 ± 18.13 ng/mL; *P* = 0.001), and DM^+^CAD^+^ (120.74 ± 21.17 ng/mL; *P* = 0.001) (Figure 3C). When compared to DM^−^CAD^+^, HA concentration was significantly higher in DM^+^CAD^−^ (*P* = 0.008) and DM^+^CAD^+^ (*P* < 0.001) with a significant difference between two diabetic groups (*P* = 0.003). Correlation of HA levels with Cascore, NCPV, and TPV revealed ρ = 0.29, *P* = 0.076, ρ = 0.30, *P* = 0.073, and ρ = 0.31, *P* = 0.063, respectively.

### Risk Factors and Molecular Markers Analysis

Analysis on risk factors and molecular data showed no significant association between sex and SOD2, LXRα, and HA (*P* = 0.85; *P* = 0.21; *P* = 0.75, respectively) as well as regarding familiarity (*P* = 0.83; *P* = 0.64; *P* = 0.55, respectively) and smoke (*P* = 0.73; *P* = 0.17; *P* = 0.49, respectively). Furthermore, no significant correlations were found between BMI and the three molecular parameters (SOD2 ρ = 0.12, *P* = 0.35; LXRα ρ = −0.11, *P* = 0.35; HA ρ = 0.19, *P* = 0.15). HA was significantly correlated with age (ρ = 0.46, *P* < 0.001), unlike SOD2 (ρ = 0.03, *P* = 0.97) and LXRα (ρ = 0.03, *P* = 0.95). HA showed also a significant association with dyslipidemia (*P* = 0.01) and diabetes (*P* = 0.01) in the study population, while statistical analysis on hypertension revealed a *P* = 0.08. On the other hand, SOD2 and LXRα were not significantly associated with the previously mentioned risk factors (SOD2 vs dyslipidemia *P* = 0.45, SOD2 vs hypertension *P* = 0.63, SOD2 vs diabetes *P* = 0.47, LXRα vs dyslipidemia *P* = 0.85, LXRα vs hypertension *P* = 0.53, and LXRα vs diabetes *P* = 0.61). Correlation between HA levels and glycemia was statistically significant (ρ = 0.73, *P* < 0.001) (Figure 3D), while no significant correlation was found between SOD2 (*P* = 0.62) and LXRα (*P* = 0.55) gene expression and glycemia.

## Discussion

In this study, we have exploited an imaging and molecular based analysis to investigate diabetic-specific CAD alterations in selected groups of patients.

Calcium score, CPV, plaque burden, and RI were significantly higher in DM^+^CAD^+^ compared to DM^−^CAD^+^. Previous studied have examined CAD and plaque features in diabetic patients by CTCA. Diabetics showed extensive coronary artery calcium deposits and, therefore, a larger atherosclerotic plaque burden with a consequent higher risk for all-cause mortality than in non-diabetic patients (5, 47–54). Gao et al. (47) found that diabetics compared to non-diabetics have higher total coronary artery calcium, a higher proportion of coronary segments with plaque and multivessel obstructive disease. In a study by Van Werkhoven et al., obstructive CAD and the number of diseased segments, with obstructive and non-obstructive plaques, were higher in diabetics than non-diabetics. Total Agatston score was higher in diabetic patients (440 ± 786 vs 195 ± 404, *P* < 0.001) (5). Khazai et al. found that segment involvement score, segment stenosis score, and total plaque score were higher in diabetics but there was no significant difference in the number of non-calcified plaque between the two groups (50). In one study by Pundziute et al., diabetics showed more diseased segments and more segments with non-obstructive CAD, but Agatston score was similar between the two groups (54). Furthermore, Chu et al. detected more calcified plaques than mixed or non-calcified plaques in diabetics. Among the different degrees of stenosis, mild narrowing was most common, and no significant difference between non-obstructive stenosis and obstructive stenosis was observed (48). In agreement with the aforementioned works, in our study, DM^+^CAD^+^ presented more diseased coronaries in terms of coronary calcium, significant stenoses, atherosclerotic burden, and extent of disease. Furthermore, we have quantified RI in diabetic patients by CTCA providing an additional prognostic value comparable only to invasive procedures such as intravascular ultrasound (55, 56). A recent study analyzed CAD features comparing hypertensive, dyslipidemic, and diabetic patients by CTCA reporting the prevalence of positive remodeling as a qualitative parameter (57).

Positive coronary arterial remodeling is a compensatory enlargement of coronary arterial lumen in response to atherosclerotic plaque formation. Histopathological studies proved that positive remodeling is associated with infiltration of inflammatory cells, expression of pro-inflammatory cytokines, and increased protease activity (58, 59). Positive remodeling is associated with vulnerable plaque and progression of atherosclerosis. High plaque burden, together with positive remodeling, means more prone to rupture plaques in diabetic patients and, therefore, a worse prognosis and a major likelihood of cardiac event occurrence.

In the last decade, a great amount of data demonstrated a complex interaction between blood cells and the arterial wall with the consequent activation of oxidative and inflammatory pathways, leading to the development of CAD.

Our results showed that the expression levels of SOD2 gene were reduced in CAD patients compared to HS, while no significant difference was found between diabetic and non-diabetic CAD subjects. Previous studies reported controversial findings for the effect of SOD2 activity relative to CAD. A recent study by Peng et al. (60) showed that plasmatic concentration of SOD1 and SOD2 was higher in CAD than in healthy control. Our findings were in line with a gene expression study performed by Abdullah et al. (61) showing a downregulation of this gene in PBMCs of angiographically confirmed CAD patients (≥50% stenosis). These data indicate that SOD2 might serve as surrogate biomarker for CAD.

Data from *in vitro* and *in vivo* models have demonstrated a key role of LXRα in the regulation of processes involved in CAD and DM such as inflammation and glucose homeostasis (62, 63). Our findings reported that LXRα gene expression was significantly upregulated in DM^+^CAD^+^ and DM^−^CAD^+^ compared to HS. Although previous study by Dahlman et al. (64) investigated the association of LXRα and DM, we demonstrated also a differential expression of this gene between DM^+^CAD^+^ and DM^−^CAD^+^ groups suggesting this parameter as a possible biological hallmark for diabetic condition. HA plasmatic concentrations showed significant difference between diabetic and non-diabetic patients with higher values in patients affected by both DM and CAD suggesting a possible additive detrimental effect on endothelial dysfunction. A significant positive correlation was found between HA levels and glycemia in our study population. Our findings were in line with previous studies, also reporting a critical role for HA in DM-related atherosclerosis (26–29, 65). In vascular dysfunction, HA triggers smooth muscle cells’ dedifferentiation, which contributes to vessel wall thickening. Furthermore, HA is able to modulate inflammation by altering the adhesive properties of endothelial cells. In hyperglycemic conditions, HA accumulates in vessels and can contribute to the diabetic complications in macro- and microvasculature (25).

However, no study has yet examined the relationship between HA levels and vascular function assessed by CTCA. Our data suggested that serum HA levels positively correlated with poor glycemic control and angiopathy and, due to the pivotal role in favoring atherogenesis, this molecule could be used as a surrogate marker of vascular function.

*In vitro* molecular analysis represents a promising tool to stratify patients for CAD risk, while *in vivo* CTCA analysis is able to identify and characterize selective diabetic coronary features. These results gain clinical relevance, considering that most patients referred to elective invasive coronary angiography for CAD suspicious are not found to have obstructive CAD (66, 67). In patients with molecular alterations suggestive for CAD, we demonstrated by CTCA specific changes of coronary plaques in diabetic patients. Moreover, recently, this imaging technique has been used to evaluate its long-term prognostic value among patients with diabetes mellitus compared with non-diabetic subjects (68).

Nevertheless, our study has some limitations: the reduced sample size has influenced the statistical power; therapeutic treatments could have affected our results; a more accurate analysis with different genomic/proteomic techniques, on a wider pool of *in vitro* markers is needed to deeply investigate molecular and imaging phenotypic interplay in diabetic CAD patients. The analyzed biomarkers are not myocardial specific CAD molecules but can be downregulated or upregulated in blood also in presence of atherosclerotic processes involving peripheral arteries and/or supra aortic vessels. Moreover, recent studies have demonstrated that diabetes can be considered a CAD equivalent condition, independently from the clinical/imaging evidences of pathology (69, 70), determining the choice of specific diabetic-related CAD biomarkers attractive.

## Conclusion

This study suggests an imaging and molecular based analysis to investigate cardiovascular alterations in diabetic patients. CTCA imaging parameters highlight a greater severity of CAD in diabetic patients. Among molecular parameters, HA is modulated by diabetic CAD-related alterations while SOD2 and LXRα are found to be more associated with CAD rather than to diabetes. Further studies are needed to better characterize the pathology and identify more specific biomarkers, also considering the complex multifactorial pathophysiology of CAD in diabetic patients.

## Ethics Statement

The study was approved and performed in accordance with the ethical standards of the institutional ethics committee (IRCCS Fondazione SDN, protocol no. 7-13) and with the Helsinki Declaration. Written informed consent was obtained from all subjects for being included in the study.

## Author Contributions

CC designed and supervised the study. TI and EF recruited subjects, performed the experiments and data analysis, and wrote the manuscript. MA, MS, and CC reviewed the manuscript. All the authors read and approved the final manuscript and agreed to its submission.

## Conflict of Interest Statement

The authors declare that the research was conducted in the absence of any commercial or financial relationships that could be construed as a potential conflict of interest.

## Abbreviations

DM, diabetes mellitus; CAD, coronary artery disease; HS, healthy subjects; DM^+^CAD^−^, diabetic non-CAD patients; DM^+^CAD^+^, diabetic patients with CAD; DM^−^CAD^+^, non-diabetic patients with CAD; CTCA, computed tomography coronary angiography; HU, Hounsfield unit; Cascore, calcium score; CPV, calcified plaque volume; NCPV, non-calcified plaque volume; TPV, total plaque volume; RI, remodeling index; SOD2, superoxide dismutase 2; LXRα, liver X receptor alpha; HA, hyaluronan; NSP, number of coronary artery segments with plaque; NCS, number of coronaries with significant stenosis.
